# Representative QRS loop of the VCG record evaluation

**DOI:** 10.3389/fphys.2023.1260074

**Published:** 2024-01-04

**Authors:** Jan Kijonka, Petr Vavra, Marek Penhaker, Jan Kubicek

**Affiliations:** ^1^ Department of Cybernetics and Biomedical Engineering, Faculty of Electrical Engineering and Computer Science, VSB—Technical University of Ostrava, Ostrava, Czechia; ^2^ Department of Surgical Studies, Faculty of Medicine of the University of Ostrava, Ostrava, Czechia

**Keywords:** digital filtering, ECG, intra-individuality, isoelectric line detection, QRS detection, QRS loop alignment, representative QRS loop, VCG

## Abstract

**Introduction:** This study proposes an algorithm for preprocessing VCG records to obtain a representative QRS loop.

**Methods:** The proposed algorithm uses the following methods: Digital filtering to remove noise from the signal, wavelet-based detection of ECG fiducial points and isoelectric PQ intervals, spatial alignment of QRS loops, QRS time synchronization using root mean square error minimization and ectopic QRS elimination. The representative QRS loop is calculated as the average of all QRS loops in the VCG record. The algorithm is evaluated on 161 VCG records from a database of 58 healthy control subjects, 69 patients with myocardial infarction, and 34 patients with bundle branch block. The morphologic intra-individual beat-to-beat variability rate is calculated for each VCG record.

**Results and Discussion:** The maximum relative deviation is 12.2% for healthy control subjects, 19.3% for patients with myocardial infarction, and 17.2% for patients with bundle branch block. The performance of the algorithm is assessed by measuring the morphologic variability before and after QRS time synchronization and ectopic QRS elimination. The variability is reduced by a factor of 0.36 for healthy control subjects, 0.38 for patients with myocardial infarction, and 0.41 for patients with bundle branch block. The proposed algorithm can be used to generate a representative QRS loop for each VCG record. This representative QRS loop can be used to visualize, compare, and further process VCG records for automatic VCG record classification.

## 1 Introduction

The development of algorithms for an automatic classification of vectorcardiographic (VCG) records for the purpose of heart disease recognition helps include VCG among the commonly used diagnostic methods. Based on the facts from recent studies [e.g., Lee et al., 1968; Simonson, 1976; Ge, 2008; Huebner et al., 2010; Romero et al., 2010; Dehnavi et al., 2011; Correa et al., 2016; Lingman et al., 2016], VCG achieves more accurate results than the standard 12-lead electrocardiographic (ECG) method. Compared to the empirically assessed 12-lead ECG, VCG diagnostics offers a quantitative description of the heart’s electrical field and of the objective view on the heart vector propagation. Thanks to the three orthogonal X, Y, and Z leads, VCG represents a suitable alternative for computerized data processing with no redundant information (Kral, 2006).

Preprocessing of a VCG record is an important initial step of the classification process commonly involving techniques of filtering, which meets the requirements for diagnostic ECG frequency bands (Kligfield et al., 2007), fiducial time instants of the QRS peak, QRS onset, and QRS end; P- and T-wave peaks, onsets, and ends; isoelectric PQ interval assessment (Pan and Tompkins, 1985; Dokur et al., 1996; Soria-Olivas et al., 1998; Vullings et al., 1998; Martínez et al., 2004; Mazomenos et al., 2012); spatiotemporal QRS loop alignment (van Alsté et al., 1986; Sörnmo, 1993; Sörnmo, 1998; Astrom et al., 2000; Vullings et al., 2013); and finally a representative P-QRS-T loops of a VCG record evaluation.

Recent studies (Sörnmo, 1998; Astrom et al., 2000; Vullings et al., 2013) propose various methods for QRS loop alignment by transformations which consist of rotation and lead-independent or lead-dependent scaling. A crucial modeling issue is, however, whether the diagnostic information of the signals is retained or becomes distorted when these transformations are applied, especially in pathological cases, where the QRS loop planarity is not retained (Schellong, 1939; Pipberger et al., 1962). Moreover, the performance of these methods is strongly dependent on signal-to-noise ratio (SNR) conditions or on *a priori* information about these transformations.

As a representative template of the QRS loop, characterizing a VCG record, theoretically any of the detected QRS loops of a record could be selected. However, a more accurate method is to evaluate an average QRS loop, where effects of heart movement during the respiration cycle and distance between surface electrodes and the heart variations, along with effects of random noises caused, e.g., by muscular activity, are minimized by the averaging. Ectopic heartbeats, if presented, should be automatically detected and excluded from the record before evaluation of the average heartbeat (Berbari et al., 1993).

Kijonka et al. (2022) focused on the fiducial points of P-QRS-T wave detection based on wavelet transform evaluated on the Physikalisch Technische Bundesanstalt (PTB) diagnostic database and validated on the Common Standards for Quantitative Electrocardiography (CSE) multilead database of 125 records of patients with various diagnoses, including healthy controls (HCs) and patients with myocardial infarction (MI), bundle branch block (BBB), and aspecific conduction defects with significant changes in the ECG image, causing a wide QRS (>120 ms). The QRS peak was evaluated correctly for all of 1,467 beats. The QRS onset and QRS end were detected with standard deviation comparable to or better than other well-known algorithms (Pan and Tompkins, 1985; SAHAMBI et al., 1997; MAZOMENOS, 2012; VULLINGS et al., 1998). The isoelectric interval was detected correctly between the P end and QRS onset for all the cases. The algorithm well-evaluated a wide QRS based on automated wavelet scale switching.

This study builds on the validated QRS loop boundaries and isoelectric coordinate detector presented in Kijonka et al. (2022) for the purpose of further signal processing—the representative QRS loop of a record evaluation technique presented here. This study deals with a suitable digital finite impulse response (FIR) filter design, including automatic notch filter design, a technique of QRS loop spatial alignment, QRS loop time-synchronization, and ectopic QRS loop elimination presented here. Compared to the QRS loop alignment techniques presented in Sörnmo (1998); Astrom et al. (2000); Vullings et al. (2013), the proposed algorithm omits transformation techniques, which could introduce a distortion in the average QRS loop of a record evaluation. Spatial and time synchronization to average the effect of heart movement during respiration, distance electrode variations, and muscular and random noises along with automatic detection to eliminate ectopic rhythms are used instead. To compare the results with those of the previous studies (Sörnmo, 1998; Astrom et al., 2000; Vullings et al., 2013), the ratio of the morphologic variability reduction before and after the proposed algorithm application was assessed separately for three diagnostic groups of HC, MI, and BBB subjects. To evaluate the signal morphologic variability, the maximum relative deviation $\delta_{\mathrm{MAX}}\left(\%\right)$ was assesed. This parameter provides us the maximum spatial distance from the average QRS loop in three signals X,Y,Z relative to the range of signals.

The proposed preprocessing algorithm was applied to VCG records of the PTB database of 58 HC, 69 MI, and 34 BBB subjects, where 1/3 of the records of each diagnostic group were used for the algorithm design. The records were 2 min long, containing approximately 120 beats for averaging. The records are sampled at 1.000 Hz (Bousseljot et al., 1995; Goldberger et al., 2000).

## 2 Materials and methods

**FIGURE 1:**
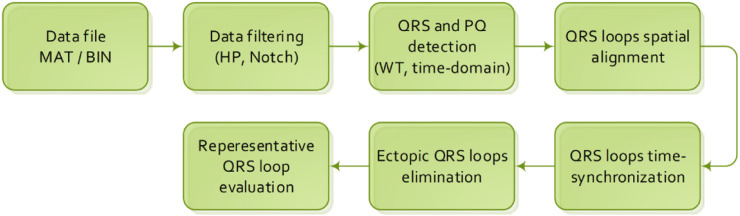
VCG preprocessing algorithm.

The initialization step of the data preprocessing algorithm (Figure 1) is loading of an input database of VCG records accompanied by an anamnesis. In case of the PTB diagnostic database, a record is stored in a MAT/BIN file, accompanied by an anamnesis HEA file.

The VCG preprocessing algorithm is described by individual steps described below in Section 2.1 to Section 2.6.

### 2.1 Data filtering

In the first step of the algorithm, as shown in Figure 1, a baseline wander and noise motion artifacts are filtered by the FIR high-pass (HP) filter with a passband cutoff frequency of 1 Hz with respect to recommendations from Kligfield et al. (2007). Other artifacts caused by electromagnetic interference (EMI) of the 50-Hz power line are removed using a notch FIR filter. This type of filter was designed for offline biosignal processing due to its linear phase and minimal distortion of the filtered signal (Marchon and Naik, 2018).

#### 2.1.1 High-pass filter design

According to the American Heart Association (AHA), the filter with a cutoff frequency $f_{c}=0.05\;\text{Hz }\left(-3\;\text{dB}\right)$ is suitable for diagnostic purposes (MEDTEQ) (Reich, 2011). This analog first-order RC filter has a non-linear phase frequency characteristic, so it significantly distorts the VCG signal up to a frequency approximately one order higher than $f_{c}$ .

The experimental measurements show that the cutoff frequency $f_{c}=0.05\;\text{Hz}$ is insufficient for motion artifact filtering for the selected database file. Therefore, HP digital FIR filters with a higher cutoff frequency are designed. According to the relative specification of the filter, the parameters for the cutoff frequencies in the passband $f_{\text{pass}}$ in the range from 0.2 Hz to 2 Hz and the cutoff frequencies in the stopband $f_{\text{stop}}=\frac{f_{\text{pass}}}{2}$ are selected. The requirement for the passband ripple $A_{\text{pass}}<0.017\;\text{dB}$ and stopband attenuation $A_{\text{stop}}>54\;\text{dB}$ is selected as well.

With respect to the requirement for $A_{\text{pass}}$ (Eq. 1) for the ripple amplitude $U_{\text{ripple}\;}<1\;\mu V$ (such a small ripple does not affect the diagnostic VCG information) and amplitude of the VCG signal $U_{\text{VKG}\;}=1\text{ mV}$ ,

$$A_{\text{pass}}<20∙\log\left(\frac{U_{\text{VKG}}+2∙U_{\text{ripple}}}{U_{\text{VKG}}}\right).$$ (1)

The requirement for $A_{\text{stop}}$ (Eq. 2) for the band-stop amplitude drift is set by $U_{\text{drift}\_\text{stop}\;}<2\;\mu V$ and the drift amplitude $U_{\text{drift}\;}=1\;\text{mV}$ (with respect to motion artifacts in the input database):

$$A_{\text{stop}}>20∙\log\left(\frac{U_{\text{drift}\;}}{U_{drift\_stop\;}}\right).$$ (2)

The filters are designed using the Parks–McClellan optimalization method. To determine the effectiveness and suitability of the equivalent FIR filter with a cutoff frequency $f_{c}=0.05\;\text{Hz}$ and four FIR filters with the threshold frequencies $f_{\text{pass}}=0.2\;\text{Hz};0.5\;\text{Hz};1\;\text{Hz};2\;\text{Hz}$ , the filters are tested in randomly selected VCG records of healthy patients and patients with myocardial infarction and bundle branch blocks, which may be negatively affected by too high cutoff frequency of the filter due to a pathologically wide QRS complex.

**FIGURE 2:**
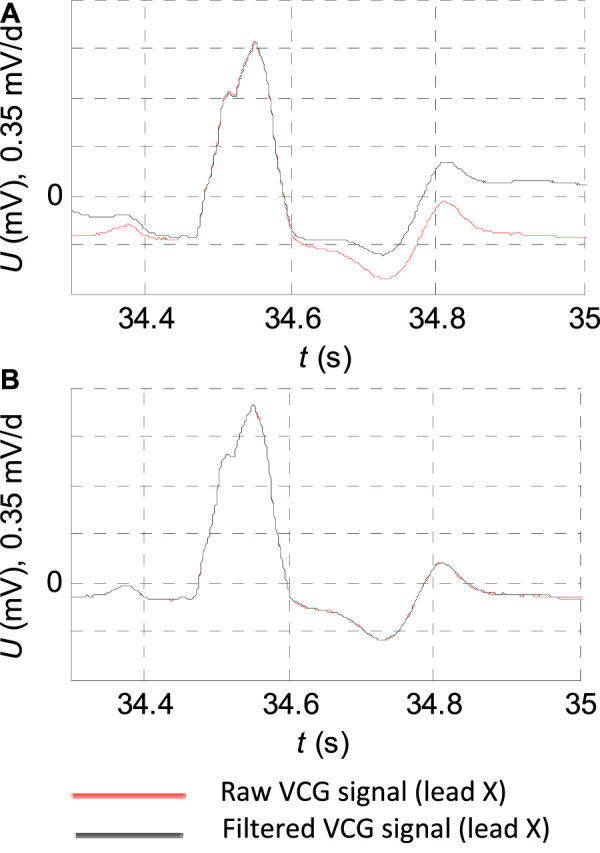
ECG record: “s0429_re” of the diagnostic PTB database of the patient with bundle branch block filtering detail. **(A)** FIR filter with the cutoff frequency $f_{\text{pass}}=2\text{ Hz}$ . **(B)** FIR filter with the cutoff frequency $f_{\text{pass}}=1\text{ Hz}$ .

In order to eliminate the isoelectric baseline fluctuation effectively, the filter with $f_{\text{pass}}=1\;\text{Hz}$ is selected. The filter with the cutoff frequency $f_{\text{pass}}=2\;\text{Hz}$ has already caused a gross distortion of the P and T waves, ST segment, and PQ segment of the records of patients with the width of the QRS complex, although the QRS complex itself has not been deformed (Figure 2).

#### 2.1.2 Automatic notch filter design

The designed notch filter is applied automatically only in the case of exceedance in the level of interference by the Eq. 3:

$$\max\left|Y\left(f_{49.5-50.5}\right)\right|>0.2∙\max\left|Y\left(f_{5\_15}\right)\right|,$$ (3)

where $\left|Y\left(f_{49.5-50.5}\right)\right|$ represents the amplitude frequency spectrum in the range 49.5 Hz – 50.5 Hz and $\left|Y\left(f_{5\_15}\right)\right|$ is the amplitude frequency spectrum in the range 5 Hz – 15 Hz .

According to the European standard EN 50160, “*Voltage characteristics of electricity supplied by public distribution systems*” is the mains frequency 50 Hz with tolerance ±0.50 Hz for 99.5 % of the time defined. The harmonic voltage cannot exceed 6 % of the fundamental frequency amplitude.

For electromagnetic interference (EMI) filtering, the FIR notch filter is designed using Parks–McClellan optimalization. The requirement for $A_{\text{stop}}>29\;\text{dB}$ is selected by keeping the mains interference amplitude $U_{50}<50\;\mu V$ . The cutoff frequency in the first and the second passbands $f_{\text{pass}1}=49.5\;\text{Hz}$ and $f_{\text{pass}2}=50.5\;\text{Hz}$ is selected considering the feasibility of the filter and to meet the conditions for the narrowest band in accordance with VCG diagnostic information preservation.

The requirement for $A_{\text{stop}}$ is fulfilled for the designed filter with the bandwidth 0.1 Hz . In the case of larger deviation from the fundamental frequency, the frequency of the notch filter $f_{\text{notch}}$ is adjusted automatically based on the signal frequency spectrum. Filtering of harmonics is meaningless since the amplitude of the interferences reached a negligible level.

### 2.2 QRS and PQ detection

One of the crucial steps in ECG analysis is to accurately detect the different waves forming the entire cardiac cycle. Most of the studies based on wavelet transformation identify almost all morphologies of ECG waveforms (Lingman et al., 2016). Especially, the wavelet transformation is worth investigating in P- and T-wave recognition (Addison, 2005; Martinez et al., 2004).

In this section, we present our previous work: a design of the QRS peak detector (detector of the R wave) including the time instants of the QRS onset and QRS end detection and the isoelectric PQ segment detection. The algorithm is based on biorthogonal wavelets since they excite various morphologies of ECGs better at different scales (Kijonka et al., 2022).

**FIGURE 3:**
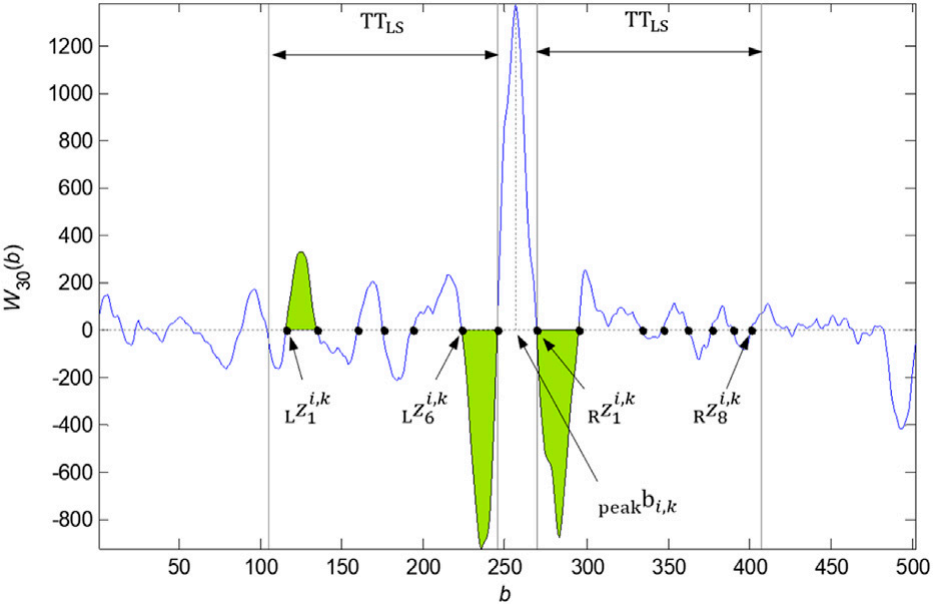
Zero points zsi,kL left to the bpeaki,k and zti,kR right to the bpeaki,k detected in the bpeaki,k neighborhood given by the parameter ${\text{TT}}_{\text{LS}}$ . The intervals between the zero points, which meet the conditions assessed (the amplitude threshold exceeding and others), are marked in green.

Here, we summarize the algorithm in eight points. The implementation with zero points and intervals detected is shown in Figure 3.

#### 2.2.1 Basics

The wavelet transform allows us to analyze nonstationary nature signals with localization in time. For analysis, the continuous form of wavelet transform (CWT) was used, described by the Eq. 4:

$$\left(W_{\psi }f\right)\left(a,b\right)=\int_{-\infty }^{\infty }{\left|a\right|}^{-1/2}f\left(t\right)\bar{\psi \left(\frac{t-b}{a}\right)}dt,$$ (4)

where a stands for a dilatation parameter, b is the translation parameter, and ψ is a mother wavelet. CWT uses sampled data, but compared to the discrete wavelet transform (DWT), it allows finer resolution. The output is a transformed signal of the same number of samples as the original. A compact and symmetric biorthogonal wavelet was used. It provides time symmetry, prevents phase shifts of the transformed signal, and complies with the shape like the detected waveforms.

#### 2.2.2 QRS peak detection

QRS peak detection is based on the wavelet transform $W_{a}^{i}\left(b\right)$ of the input signals $i\in I=\left\{1,2,3\right\}$ corresponding to X,Y,Z signals, on scale a , where the samples of the record $b\in B=\left\{1,\ldots ,N\right\}$ , where N is the total number of samples of the signal. For the detection, the scale a=30 is used, which corresponds with the biorthogonal wavelet of pseudo-frequency approximately 30 Hz . The appropriate scale for QRS detection is determined experimentally based on the QRS frequency band. In the next step, the occurrences of the creation of QRS peaks are calculated. These sets are arranged based on exceedance of the amplitude threshold of the transformed signal and based on the specified maximum heart rate. From each set, just one time instant according to established rules was selected. It corresponds to the expected R peak wave.

#### 2.2.3 QRS onset and QRS end detection

The QRS onset and offset detection is based on zero crossing of $W_{a}^{i}\left(b\right)$ . Zero points in the neighborhood of the local maxima of the function $W_{a}^{i}\left(b\right)$ (Figure 3) are searched individually for each signal and each QRS detected. The zero points of the QRS onset and QRS end are determined based on the conditions set for exceeding the amplitude threshold of $W_{a}^{i}\left(b\right)$ between the zero-point intervals, interval lengths, and the sequence of suitable or unsuitable intervals (intervals that meet defined conditions) (Kijonka et al., 2022). The QRS onset and QRS end are then adjusted according to the $W_{a}^{i}\left(b\right)$ signal shape in the preceding or following interval.

#### 2.2.4 Wide QRS onset and QRS end adjustment

In some cases, e.g., blockades, a wide QRS might occur. The previous parameters of the defined neighborhood of ${\text{TT}}_{\text{LS}}$ and a=30 scale would be inadequate for the QRS onset and QRS end detection. The algorithm sets the wide QRS based on the conditions (Kijonka et al., 2022) and adjusts the wide QRS onset or QRS end. The wide QRS is evaluated based on the energy percentage of the wavelet coefficient rate in the neighborhood of the original QRS onset and QRS end in the scales a=70  and a=120 , respectively, and the percentage of energy in these scales on the window of width given by the TTLS1 parameter. The QRS onset and QRS end adjustment is based on the $W_{a}^{i}\left(b\right)$ zero crossing on scale a=70 , while a similar procedure as in QRS onset and QRS end detection is maintained.

#### 2.2.5 QRS onset and QRS end adjustment by slope

The adjustment of QRS onset and QRS end using the linear regression is based on the calculation of the slope at the temporal search window applied to the input signal in the area before the QRS onset or after the QRS end detected previously. The QRS onset or QRS end is shifted to the point that meets the specified threshold for the line slope (Kijonka et al., 2022) in the temporal search window.

#### 2.2.6 PQ detection

The PQ segment detection using linear regression is based on finding a minimum slope on the temporal search window of the input signal in the neighborhood of the QRS onset. The time window with width 10 ms is selected experimentally (Kijonka et al., 2022).

#### 2.2.7 QRS onset and QRS end alignment between X, Y, and Z signals of a VCG record

The QRS onset and QRS end alignment between the X,Y,Z signals is determined based on conditions for exceeding the distances of the detected QRS onsets or ends. The maximum distance between the QRS onsets is set by the ${\text{TT}}_{\text{BS}}$ parameter (Kijonka et al., 2022). In the case of an exceeding threshold, the further point is shifted to the mean value of the remaining two points. This process increases the robustness of the algorithm, and it is performed for the correct PQ segment detection and the correct QRS loop boundary detection in the case of erroneous QRS onset or end detection in some of the three VCG signals.

#### 2.2.8 QRS loop boundary detection

The QRS boundaries of the record are given by the boundL and boundR , where boundL is the left bound of the QRS loop and boundR is the right bound of the QRS loop. The values of the boundaries (in samples) indicate the distance between the synchronization wave and the left or right QRS loop bound. The detected QRS in the lead X is marked as the synchronization wave. The boundL and boundR represent the constants for all the QRS loops of the record for the dominant length of the QRS loop. The boundL and boundR parameters are computed using the Eqs. 5, 6:

boundL=medk=1,…,pmaxi∈I r1,k−maxPQi,k, (5)

boundR=medk=1,…,pmax i∈Ir1,k−si,k, (6)

where k is the sequence number of the QRS detected, i is the index of the signal, $r_{1,k}$ is the synchronization wave, ${\text{PQ}}_{i,k}$ is the set of points of the PQ interval, $s_{i,k}$ is the QRS offset, and $med\left(.\right)$ stands for the median value.

### 2.3 QRS loop spatial alignment

The isoelectric baseline detection is one of the most important steps in VCG preprocessing for the purpose of quantitative description by the features, especially by the features describing the P-, QRS, and T-loop spatial location. These loops should have the initial point in the origin of the coordinate system; thus, the instantaneous magnitude of the vector given by the three coordinates X,Y,Z should be 0 in the beginning of each heart action. It should be executed in the PQ and ST segments for the non-pathological cases. As the most suitable interval for the zero-heart electrical activity indication, the PQ segment appears suitable also for most of the pathological cases. The PQ intervals detected for the three X, Y, and Z VCG leads create the isoelectric coordinates of the QRS loop. The detection of the PQ intervals and QRS bounds is performed in the presented work by the methods (see Section 2.2) described in more detail in the previous work (Kijonka et al., 2022).

The correction on the isoelectric baseline is computed for each VCG signal and each QRS complex detected, by the Eq. 7:

$${\mathrm{ISO}}_{i,k}=\underset{\min\left({PQ}_{i,k}\right)<b<\;\max\left({PQ}_{i,k}\right)\;}{\mathrm{mean}}\left(f^{i}\left(b\right)\right)$$ (7)

where k is the sequence number of QRS, i is index of the signal, $f^{i}\left(b\right)$ is the input signal, b is the sample of the signal, ${\text{PQ}}_{i,k}$ is the set of the points of the PQ interval, and $\text{mean}\left(\right)$ represents the mean value.

Each detected QRS complex of the record with the sequence number $k=\left\{1,\ldots ,p\right\}$ of the signal $i=\left\{1,2,3\right\}$ bordered by the boundL and boundR is shifted by the voltage level ${\text{ISO}}_{i,k}$ so that the corresponding QRS loops are shifted in all three coordinates.

In the cases where the ${\text{ISO}}_{i,k}$ is not correctly detected for all the QRS, the other correction method by the Eq. 8:

ISO_Bi,k=fir1,k−boundL (8)

is used. Based on this relation, the isoelectric baseline ${\text{ISO}\_B}_{i,k}$ is computed only from one point of the left QRS bound. This method is used in few number of cases, especially for the MI patients (see Figure 5).

### 2.4 QRS loop time synchronization

An objective of the QRS loop time synchronization is to move individual heartbeats of a record to a common time-synchronization mark. As the time-synchronization mark, the QRS peak detected in the signal X was selected. For the optimal alignment of the individual heartbeats, a method of root mean square error minimization by individual heartbeat (three signals X, Y, and Z) time shifting, where the individual heart beats are shifted relative to the median heartbeat, is proposed.

**FIGURE 4:**
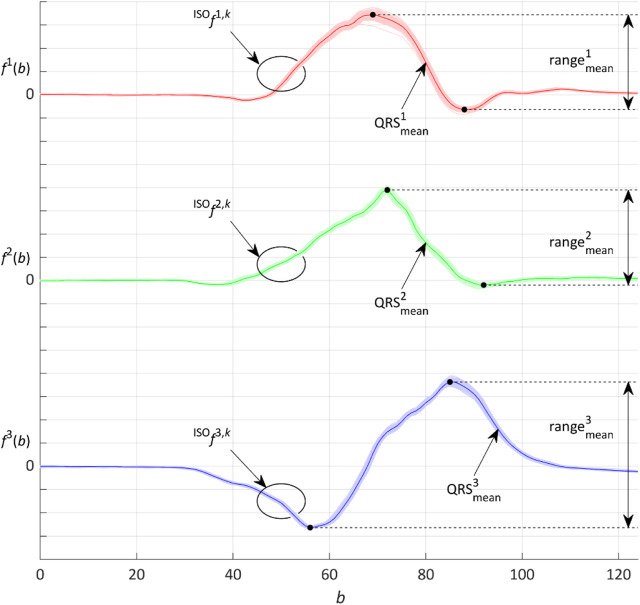
Representative QRS loop given by the three X,Y,Z signals marked as ${\text{QRS}}_{\text{mean}}^{i}$ and all the QRS of each signal corrected by ${\text{ISO}}_{i,k}$ marked as fISOi,k , where $k\in \left\{1,\ldots ,p\right\}$ and p is the number of QRS detected in the signal of index i . The range of ${\text{QRS}}_{\text{mean}}^{i}$ is marked as ${\text{range}}_{\text{mean}}^{i}$ .

All the detected QRS loops adjusted by the isoelectric coordinates (three detected isoelectric levels) are temporally aligned by the method of minimalization of the mean quadratic error from the median for the moving parameter τ . The QRS loop alignment is performable in the timescale by the overlapping method (Figure 4).

The minimalization process and the $\tau_{k}$ parameter finding for the each QRS loop k by the Eqs. 9–12:

∀i find median:

QRSmedi=medk∈1,…,p fISOi,k, (9)

and the range of the median QRS:

$${\text{range}}_{\text{med}}^{i}=\max\left({\text{QRS}}_{\text{med}}^{i}\right)-\max\left({\text{QRS}}_{\text{med}}^{i}\right),$$ (10)

where fISOi,k is the QRS detected corrected by the ${\text{ISO}}_{i,k}$

∀τ we define matrix Mτ of the mean square deviations of the function fISOi,k from ${\text{QRS}}_{\text{med}}^{i}$ , where each element mi,kτ of the matrix Mτ is given by the Eq. 11:

mi,kτ=mean fISOi,kb+τ−QRSmedi2rangemedi, (11)

where τ is the moving parameter.

Finally, the τ for each k is calculated for the mean quadratic error minimalization by the Eq. 12:

τk=minτ=−8,…,8maxk=1,…,pmi,kτ, (12)

where the moving parameter τ is chosen in the range −8 – 8 samples. These limits were chosen experimentally based on maximum variations in QRS peak detection in pathological cases. Theoretically, higher limits of $\tau_{k}$ could be selected for extremely variable signals, which will cause an increase in the algorithm evaluation time. The most probable value of $\tau_{k}$ calculated to the mean quadratic error minimalization was in the range of −2 to 2 samples ( −2 to 2 milliseconds).

The process according to Eqs. 9–12 repeats for the aligned QRS loops by the selected number of iterations. The greater the number of iterations is, the more precise the calculation of the median and of the mean quadratic deviations from the median can be achieved. A number of three iterations was chosen as an optimal value, where further increasing of iterations had a negligible effect on the mean quadratic deviation reduction.

### 2.5 Ectopic QRS loop elimination

The ectopic QRS loops can be presented in the record, e.g., due to the presence of ventricular extrasystoles, arrhythmias, or artifacts. To evaluate the representative QRS loop of the record, these ectopic QRS loops cannot be considered for the calculation. The ectopic QRS loops were identified by the extreme observation (outlier) method such that ∀i,k , the maximum square deviation, is computed (Eq. 13). Similar statistical methods for the outlier detection in biosignals were also used in Cipra et al. (1990).

devi,k=max fISOi,k−QRSmeani2rangemeani, (13)

where ${\text{QRS}}_{\text{mean}}^{i}$ and ${\text{range}}_{\text{mean}}^{i}$ stand for the mean values calculated analogously by Eqs. 9, 10.

Extreme observations with the interquartile range (IQR) ∀i can be calculated by the Eq. 14:

$${\mathrm{dev}}_{i,k}<x_{0.25}-3\mathrm{IQR}\cup {dev}_{i,k}>x_{0.75}+3\mathrm{IQR}.$$ (14)

Then, ${\text{dev}}_{i,k}$ is the extreme observation.

All the k QRS loops, for which at least one signal i meets Eq. 14, are excluded within the representative QRS loop of the record calculation.

### 2.6 Representative QRS loop evaluation

The output of the algorithm for VCG signal preprocessing is the representative QRS loop of a record calculated as the average of the QRS loops of the three X,Y,Z signals, moved on the voltage axes to the isoelectric coordinates, aligned in the time axes and treated out of the outliers.

To assess the signal morphologic variability before and after performing the presented methods of spatial alignment, time synchronization, and ectopic QRS elimination, the maximum relative error $\delta_{\mathrm{MAX}}$ parameter was computed by the Eq. 15:

δMAX=maxfISOi,k−QRSmeani∙100rangemeani, (15)

where $\delta_{\mathrm{MAX}}$ computes the maximum spatial distance from the average QRS loop in the three signals X,Y,Z relative to the range of signals. The maximum spatial distances of individual QRS loops from the average QRS loop are clearly visible in Figure 4, where all the QRS loops of a record are shown by the overlapping display method (brighter color) and calculated mean QRS loop—representative QRS loop of the record is shown in a darker color. A signal with the largest deviation from the mean indicates the maximum error.

The results of $\delta_{\mathrm{MAX}}$ for the records of healthy controls (HC), MI patients, and BBB patients are shown in Table 1. Low values of $\delta_{\mathrm{MAX}}$ indicate low intra-individual variability and, therefore, a more accurate calculation of the representative QRS loop of the record.

**TABLE 1:** Summarization of $\delta_{\mathrm{MAX}}$ for the HC, MI and BBB diagnoses.

	$\delta_{\mathrm{MAX}–1}$ (%)	$\delta_{\mathrm{MAX}–2}$ (%) reduction factor (−)	$\delta_{\mathrm{MAX}–3}$ (%) reduction factor (−)	Extreme observations (%)
**HC**	19.2	14.6	12.2	2.9
		0.24	0.36	
**MI**	31.1	22.2	19.3	2.9
		0.29	0.38	
**BBB**	29.1	20.1	17.2	4.2
		0.31	0.41	

$\delta_{\mathrm{MAX}\_1}$ —without ttime-synchronization, extreme observations not excluded.

$\delta_{\mathrm{MAX}\_2}$ —without time-synchronization, extreme observations excluded.

$\delta_{\mathrm{MAX}\_3}$ —time-synchronized, extreme observations excluded.

The $\delta_{\mathrm{MAX}}$ parameter also plays an important role in determining the patient’s condition in long-term patient monitoring, where the significant changes in intra-individual variability [changes in $\delta_{\mathrm{MAX}}$ greater than 10% (Laufberger, 1980)] point to the deterioration or improvement of the patient’s state.

## 3 Results

The maximum relative error $\delta_{\mathrm{MAX}}$ is evaluated for individual diagnostic groups of 58 HC, 69 MI, and 34 BBB subjects (Figure 6). A significant reduction of the $\delta_{\mathrm{MAX}}$ is achieved by the ectopic QRS elimination described in Section 2.5. Further reduction is achieved by the time synchronization technique presented in the Section 2.4 in combination with the ectopic QRS elimination, while the percentage of the detected ectopic QRS is preserved or reduced.

A summary of the results of $\delta_{\mathrm{MAX}}$ is presented in Table 1, where the $\delta_{\mathrm{MAX}}$ is evaluated for individual diagnostic groups of HC, MI, and BBB subjects. The lower value of $\delta_{\mathrm{MAX}}$ and, thus, probably, the lower intra-individual variability are evaluated in healthy subjects. A relatively high average reduction factor of 0.38 for all observed diagnostic groups is achieved, without accompanying transformations methods used in previous studies (Sörnmo, 1998; Vullings et al., 2013).

**FIGURE 5:**
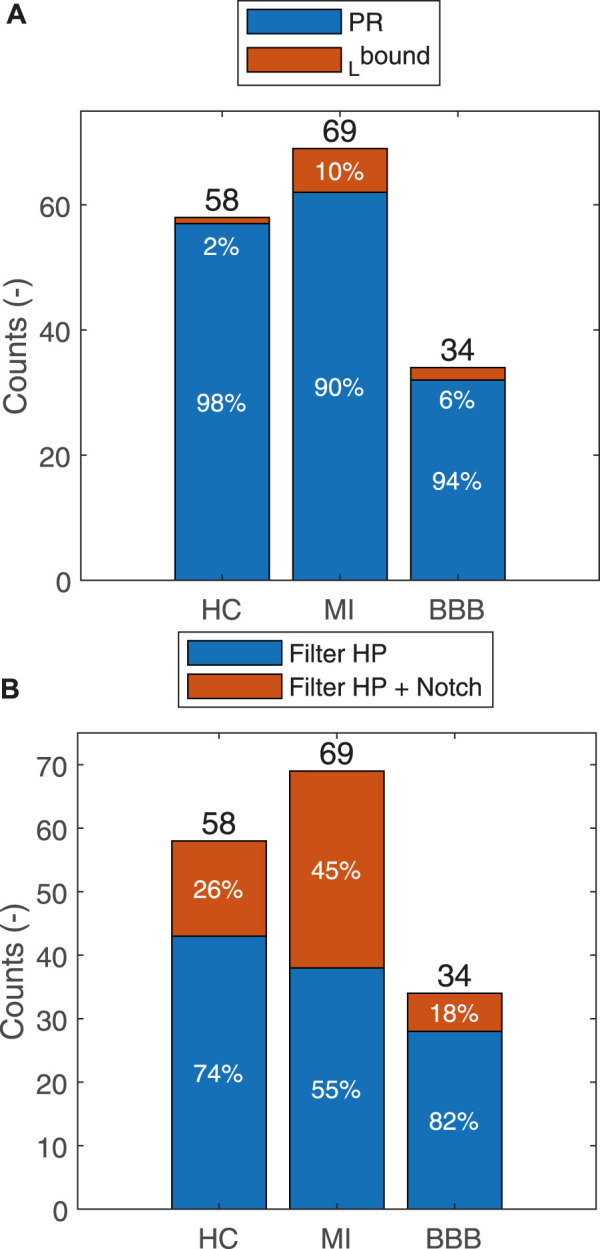
Summarization of the usage of the HP filter or HP filter in combination with the notch filter for each group for the HC, MI, and BBB diagnoses **(A)**. Isoelectric baseline by ${\text{ISO}}_{i,k}$ (PR) or by ${ISO\_B}_{i,k}$ (_L_bound) usage for each diagnostic group **(B)**.

**FIGURE 6:**
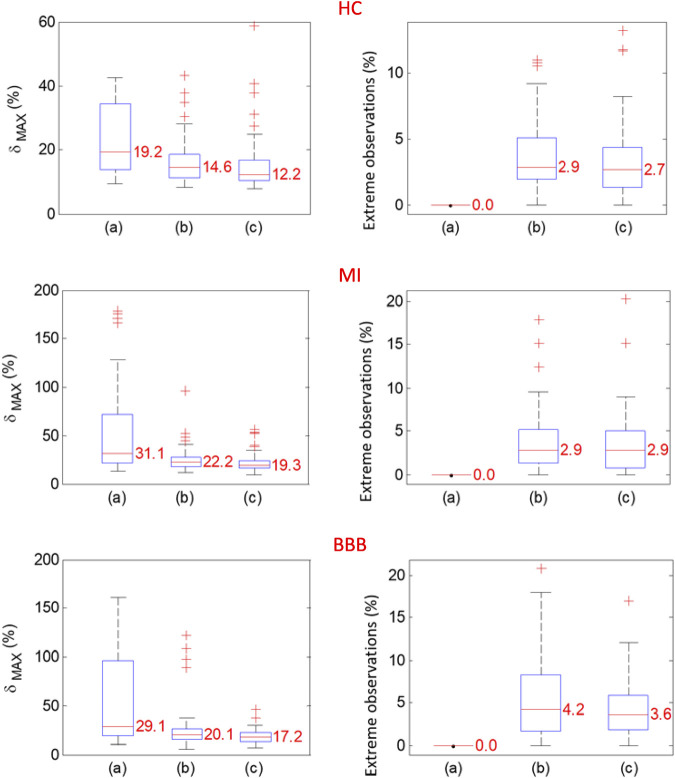
Probability distributions of $\delta_{\mathrm{MAX}}$ (%) and extreme observations (%) for the not time-synchronized QRS with the extreme observation not excluded (a), or for the not time-synchronized QRS with the extreme observation excluded (b), or for time-synchronized QRS with the extreme observation excluded (c). The calculations are evaluated for the group of HC (top), MI (middle) and BBB (bottom) subjects diagnoses.

For most of the records, only the HP filter is used. The automatically selected 50-Hz notch filter according to the Eq. 3 is especially used in the MI case (45%), subsequently in HC (26%), and least in BBB (18%) (Figure 5). For majority of the records, the isoelectric baseline detection is used by Eq. 7. The isoelectric baseline detection according to Eq. 8 is only used in the case of artifacts presented in the processed signal, which made it impossible to detect the PQ segments identically for all the QRS. The indicator of this state is also observable by a higher level of $\delta_{\mathrm{MAX}}$ (Figure 6).

## 4 Discussion

The automatic classification of a VCG record requires data preprocessing of three X, Y, and Z orthogonal leads involving algorithms for the onset and end of individual P-QRS-T loop detection. A slow baseline wander requires that the origin of a VCG loop is translated to its isoelectric coordinates before further data processing (Kijonka et al., 2022). Loop translation is considered a part of the data preprocessing used for VCG loop alignment (Sörnmo, 1998; Vullings et al., 2013). Application of these methods allows for comparison of the translated VCG loops of a single record or a comparison between different records and is substantial for intra-individual variability of a record assessment (Penhaker, 2014) for further VCG processing and VCG feature extraction considering the topological arrangement of a VCG loop (Laufberger, 1980; Le et al., 2013). To compare multiple spatially aligned QRS loops with QRS onset, QRS peak and QRS end are detected, and the QRS loops should be first time-synchronized. The best alternative for time synchronization is using the most accurately detected time instant, that is represented by the QRS peak (e.g., in the X lead). However, due to morphologic variability caused in particular by respiration-induced movements of the heart and variability in physiological origin, the QRS loops synchronized by the QRS peak still have falsely high intra-individual variability. By applying the multipass time-synchronization method presented in this study, the QRS loops are synchronized by small time shifts (±8 ms) relative to the original synchronization of the QRS peak to minimize the maximum relative error. A relatively high reduction factor of the morphologic variability is achieved. The beat-to-beat amplitude changes caused by respiration cycles and white noise are averaged in the resulting representative QRS loop of a record, where the impact of additional geometric transformation methods (Sörnmo, 1998; Astrom et al., 2000; Vullings et al., 2013) would have a negligible effect on the resulting average curve.

## 5 Conclusion

The methods of VCG signal preprocessing to compute a representative QRS loop of a VCG record evaluation were presented and applied in the analysis of VCG records from the diagnostic PTB database of 58 healthy subjects, pathological cases of 69 MI subjects, and 34 BBB subjects. Relatively small intra-individual variability was measured after spatial alignment, and time synchronization implemented by algorithms was presented in this study. The maximum relative deviation of 12.2% for HC, 19.3% for MI, and 17.2% for BBB diagnostic groups was evaluated. The variability was reduced by a factor of 0.36 for HC, 0.38 for MI, and 0.41 for BBB after QRS time synchronization and ectopic QRS elimination were performed. The presented methods of the template QRS loop of a VCG record evaluation can better differentiate between morphologies of healthy and pathological subjects of individual diagnostic groups and different degrees of disability. Application of the proposed algorithm on the other databases of VCG records is expected based on the usage of the validated method of the fiducial point of the P-QRS-T wave detection.

## Data Availability

Publicly available datasets were analyzed in this study. These data can be found at: Xingwen Fu, 12 August 2021, “ptb-diagnostic-ecg-database-1.0.0,” IEEE Dataport, doi: https://dx.doi.org/10.21227/zx3d-d450.
